# Collective assessment of antimicrobial susceptibility among the most common Gram-negative respiratory pathogens driving therapy in the ICU

**DOI:** 10.1093/jacamr/dlaa129

**Published:** 2021-02-19

**Authors:** Pamela A Moise, Marcela Gonzalez, Irina Alekseeva, Diego Lopez, Brune Akrich, C Andrew DeRyke, Wei-Ting Chen, Jacqueline Pavia, Brandon Palermo, Meredith Hackel, Mary Motyl

**Affiliations:** 1 Merck & Co., Inc, Kenilworth, NJ, USA; 2 MSD Spain, Madrid, Spain; 3 MSD Dubai, Dubai, United Arab Emirates; 4 MSD France, Puteaux, France; 5 MSD Taiwan, Taipei, Taiwan; 6 MSD Colombia, Bogota, Colombia; 7 IHMA, Schaumburg, IL, USA

## Abstract

**Objectives:**

To describe the pathogen predominance and to evaluate the probability of covering the most common Gram-negative pathogens collectively in both empirical and early adjustment prescribing scenarios in ICU patients with respiratory infections.

**Methods:**

Data were collected from an international cohort of hospitals as part of the SMART Surveillance Program (2018). Susceptibility testing (mg/L) was performed by broth microdilution methods.

**Results:**

7171 Gram-negative respiratory isolates from adult ICU patients across 209 hospitals from 56 different countries were studied. Overall, the most common ICU respiratory pathogens isolated were *Pseudomonas aeruginosa* (25%), *Klebsiella pneumoniae* (18%), *Acinetobacter baumannii* (14%), and *Escherichia coli* (11%), with inter-regional differences among these pathogens. Among Enterobacterales, 36% were ESBL positive. When the collective susceptibility profile of this set of pathogens (*P. aeruginosa* plus Enterobacterales; comprising 78% of all organisms isolated) was performed, ceftolozane/tazobactam (84%), followed by meropenem (81%), provided the most reliable *in vitro* activity in the empirical prescribing scenario compared with other β-lactam antibiotics. *P. aeruginosa* co-resistance was common among first-line β-lactam antibiotics. If *P. aeruginosa* was non-susceptible to piperacillin/tazobactam, less than one-third were susceptible to meropenem or ceftazidime. In contrast, ceftolozane/tazobactam offered *in vitro* coverage in over two-thirds of these resistant pathogens.

**Conclusions:**

Ceftolozane/tazobactam demonstrated high cumulative susceptibility levels and *in vitro* activity in both empirical and adjustment antibiotic prescribing scenarios. High frequency of co-resistance undermines reliable coverage for Gram-negative pathogens already resistant to first-line agents. Ceftolozane/tazobactam would offer additional coverage in this setting.

## Introduction

Empirical antibiotic therapy selection is based upon an integration of most likely pathogens and local antimicrobial susceptibility patterns, superimposed on clinical characteristics such as severity of illness and host comorbidities.^1–3^ Directed therapy follows the results of susceptibility testing, which either is de-escalation to narrow-spectrum regimens in cases of a susceptible pathogen or, in the unfortunate cases of antibiotic treatment failure and/or drug resistance, a need to escalate therapy. The objectives of this study were to describe collectively the predominant pathogens and their accompanying antimicrobial susceptibility in order to (i) evaluate the probability of appropriate empirical antimicrobial coverage of different regimens when treating Gram-negative respiratory infections in the ICU and (ii) determine the susceptibility to common alternative therapies during early adjustment antibiotic prescribing scenario (i.e. when a patient is not responding to first line therapies, casting suspicion on the infecting organism’s susceptibility to the prescribed antibiotic).

## Methods

**Figure 1.**
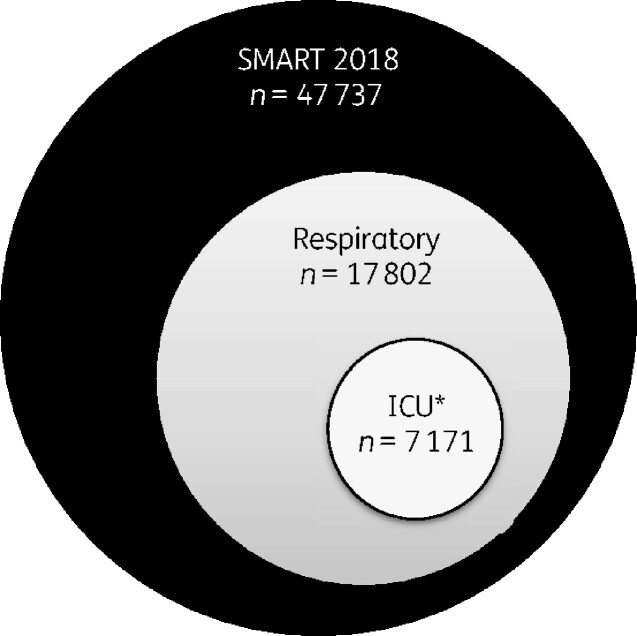
Source of clinical isolates. *Excluding paediatric ICUs.

Data were collected from an international cohort of hospitals as part of the SMART (Study for Monitoring Antimicrobial Resistance Trends) Surveillance Program. A total of 47 737 Gram- negative isolates were submitted (one per patient) to the SMART surveillance program in 2018. Each participating hospital was asked to submit up to 100 consecutive clinically relevant Gram-negative bacilli (one isolate per patient episode) from lower respiratory tract specimens, up to 50 isolates from urinary tract specimens, up to 50 isolates from intra-abdominal specimens, and up to 50 isolates from bloodstream specimens. Gram-negative respiratory isolates submitted from adult patients with respiratory infections in the ICU were included in this study (see also Figure 1).

### Ethics

The study [Protocol No. IHMA 2992 (SMART 2018)] was done in accordance with principles of Good Clinical Practice and in accordance with accepted scientific practice for this type of study. The study was approved by the institutional review boards at each participating centre. All institutions waived the requirement for obtaining informed consent.

### Susceptibility testing

Susceptibility testing (MIC, mg/L) was performed by broth microdilution methods using CLSI recommendations. Categorical interpretation of susceptibility to antimicrobial agents was defined according to the 2020 CLSI M100. For ceftolozane/tazobactam, *Pseudomonas aeruginosa* breakpoints were used to interpret MICs for *Acinetobacter baumannii*. The CLSI ESBL-phenotypic criteria for epidemiological detection of ESBL-producing organisms were used for Enterobacterales and was defined as an MIC value ≥2 mg/L for ceftriaxone.

### Data analysis and definitions

Probability of adequate antimicrobial coverage in treating respiratory tract infections in the ICU were considered for empirical prescribing and early adjustment prescribing scenarios. The empirical prescribing scenario analysis evaluated the probability of various available empirical treatments to simultaneously cover the majority of the most likely infecting pathogens (i.e. collective susceptibility). The early adjustment scenario assumed lack of a clinical response in patients who had isolates resistant to the chosen antibiotic, and calculated the likelihood of activity of one of the other agents, assuming the patient had been escalated to this agent. Statistical analysis was performed using EpiInfo.

## Results

### Isolate characteristics

**Figure 2.**
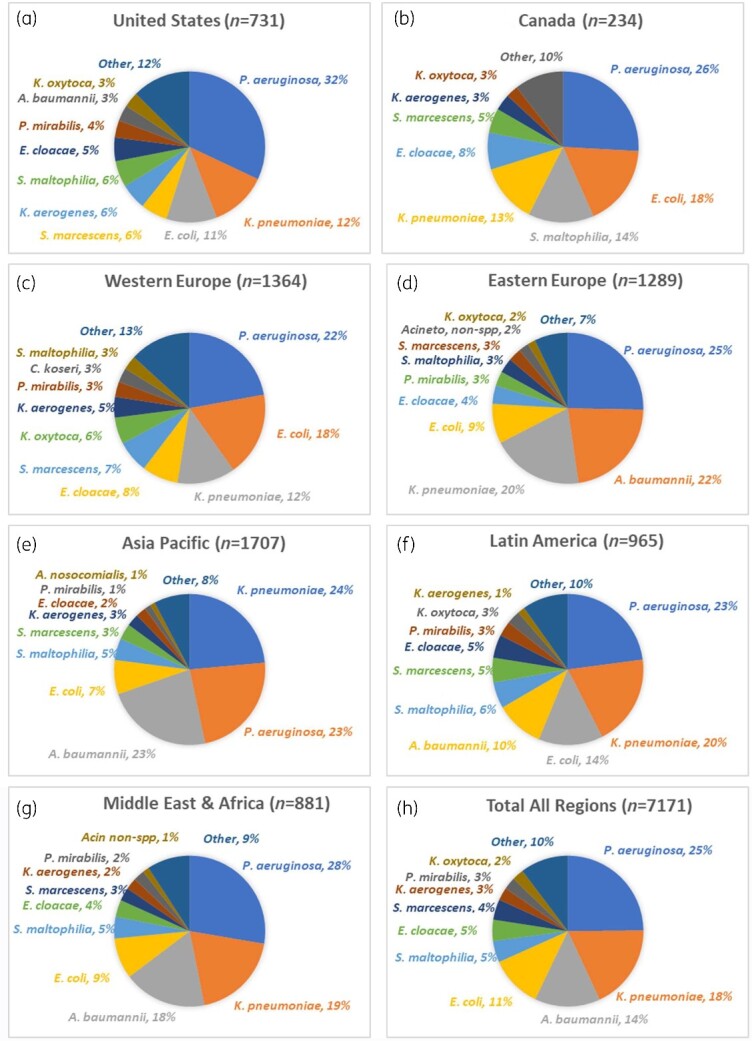
ICU respiratory pathogens, overall and by geographical region (2018).

This study included 7171 Gram-negative respiratory isolates from adult ICU patients across 209 hospitals from 56 different countries (Figure 1), including the US (*n = *731, 10%), Canada (*n = *234, 3%), Eastern Europe (*n = *1364, 19%), Western Europe (*n = *1289, 18%), Asia Pacific (*n = *1707, 24%), Latin America (*n = *965, 13%), and Middle East/Africa (*n = *881, 12%) (Figure 2). The breakdown of isolate contribution from western European countries included: Belgium (86); France (219); Germany (337); Italy (190); Portugal (76); Spain (189); Sweden (11); Switzerland (59); and the United Kingdom (122). Eastern European countries included: Croatia (108 isolates); Czech Republic (34); Georgia (84); Greece (58); Hungary (117); Latvia (38); Lithuania (154); Poland (72); Romania (79); Russia (179); Serbia (132); Slovenia (57); Turkey (180); and Ukraine (72). Asia Pacific region countries included: Australia (170); China (427); Hong Kong (39); India (127); Japan (31); Korea, South (126); Malaysia (135); New Zealand (42); Philippines (42); Taiwan (273); Thailand (161) and Vietnam (134). Latin America included: Argentina (118 isolates); Brazil (208); Chile (73); Colombia (96); Ecuador (76); Guatemala (73); Mexico (159); Panama (45); Puerto Rico (50); and Venezuela (67). The Middle East and Africa region included: Israel (162 isolates); Jordan (31); Kenya (138); Kuwait (77); Lebanon (63); Morocco (108); Qatar (53); South Africa (97); and Tunisia (142).

Overall, the four most-common ICU respiratory pathogens isolated were *P. aeruginosa* (*n = *1783, 25%), *Klebsiella pneumoniae* (*n = *1307, 18%), *A. baumannii* (*n = *1005, 14%), and *Escherichia coli* (*n = *803, 11%), with inter-regional differences among these pathogens noted (Figure 2). Among Enterobacterales, 36% (1380/3826) were ESBL-positive by phenotypic testing.

### Empirical prescribing scenario

Table 1 provides the probability of appropriately covering the most common respiratory tract infection Gram-negative pathogens from ICU patients. Among *P. aeruginosa*, ceftolozane/tazobactam displayed the highest susceptibility at 87%. In comparison, the susceptibility rates for piperacillin/tazobactam, meropenem and ceftazidime showed a very tight range, from 62% to 66%. When considering Enterobacterales, meropenem and ceftolozane/tazobactam displayed the highest activity at 91% and 83% susceptible, respectively, with activity varying among the subspecies. In this data set, all *P. aeruginosa* plus Enterobacterales comprised over 78% (5609/7171) of all Gram-negative bacteria. When the collective susceptibility profile of this set of pathogens (*P. aeruginosa* plus Enterobacterales) was calculated, ceftolozane/tazobactam (84%), followed by meropenem (81%), provided the most reliable *in vitro* activity in the empirical prescribing scenario compared with other β-lactam antibiotics (Table 1). Susceptibility rates for *A. baumannii* were only 12% for ceftolozane/tazobactam and meropenem, and 11% and 10% for ceftazidime and piperacillin/tazobactam respectively. When considering empirical monotherapy choices for *P. aeruginosa* plus Enterobacterales plus *A. baumannii*, ceftolozane/tazobactam remained the most reliable agent compared with meropenem, ceftazidime and piperacillin/tazobactam (Table 1).

**Table 1.** Probability of empirical coverage of most common Gram-negative ICU respiratory pathogens^a^

	Percentage susceptible (*n*/*N*)			
ICU respiratory pathogen	C-T	TZP	MEM	CAZ
*P. aeruginosa*	**87.1%**	62.4%	61.6%	66.0%
(*n = *1783)	(1553/1783)	(1113/1783)	(1099/1783)	(1176/1783)
*K. pneumoniae*	69.4%	61.8%	**78.3%**	51.1%
(*n = *1307)	(907/1307)	(808/1307)	(1023/1307)	(668/1307)
*E. coli*	96.0%	85.9%	**98.3%**	73.3%
(*n = *803)	(771/803)	(690/803)	(789/803)	(589/803)
All Enterobacterales	83.0%	77.5%	**90.6%**	69.0%
(*n = *3826)	(3177/3826)	(2965/3826)	(3468/3826)	(2639/3826)
*P. aeruginosa+K. pneumoniae+E. coli*	**83.0%**	67.1%	74.7%	62.5%
(*n = *3893)	(3231/3893)	(2611/3893)	(2911/3893)	(2433/3893)
All *P. aeruginosa+*Enterobacterales	**84.3%**	72.7%	81.4%	68.0%
(*n = *5609)	(4730/5609)	(4078/5609)	(4567/5609)	(3815/5609)
*A. baumannii*	**11.8%**	9.6%	11.6%	11.2%
(*n = *1005)^b^	(119/1005)	(96/1005)	(117/1005)	(113/1005)
All *P. aeruginosa*+Enterobacterales+*A. baumannii*	**73.3%**	63.1%	70.8%	59.4%
(*n = *6614)^b^	(4819/6614)	(4174/6614)	(4684/6614)	(3928/6614)

Abbreviations: CAZ, ceftazidime; C-T, ceftolozane/tazobactam; MEM, meropenem; TZP, piperacillin/tazobactam; S, susceptible.

a The highest percentage susceptibility for a given row (pathogen) is indicated in bold.

b CLSI does not define *A. baumannii* breakpoints for C-T, thus we utilized the breakpoints available for *P. aeruginosa* in the analysis for comparison only. The clinical relevance of these breakpoints is not known.

### Early adjustment prescribing scenario

Data were also analysed comparing the *in vitro* activity of these agents in the adjustment prescribing scenarios. For instance, when patients with *P. aeruginosa* pneumonia fail to improve on initial empirical therapy, clinicians frequently escalate therapy, commonly to a carbapenem, while awaiting final susceptibility testing results. However, these data demonstrate *P. aeruginosa* co-resistance to be common among first-line β-lactam antibiotics (Table 2). For example, if *P. aeruginosa* was non-susceptible to traditional first-line β-lactams, such as piperacillin/tazobactam, less than one-third were susceptible to meropenem or ceftazidime. Hence, switching to another commonly prescribed antibiotic would offer limited additional coverage. In contrast, switching to ceftolozane/tazobactam would offer significant additional coverage in over two-thirds of these non-susceptible pathogens.

**Table 2.** Probability of coverage for *P. aeruginosa* in ICU pneumonia when non-susceptible to first-line β-lactam antibiotics^a^

	Percentage susceptible (*n*/*N*)			
Pathogen and β-lactam NS phenotype	C-T	TZP	MEM	CAZ
*P. aeruginosa*	**87.1%**	62.4%	61.6%	66.0%
(*n = *1783)	(1553/1783)	(1113/1783)	(1099/1783)	(1176/1783)
TZP NS *P. aeruginosa*	**67.8%**	0	26.7%	15.2%
(670/1783, 38%)	(454/670)		(179/670)	(102/670)
MEM NS *P. aeruginosa*	**68.1%**	28.2%	0	34.9%
(684/1783, 38%)	(466/684)	(193/684)		(239/684)
CAZ NS *P. aeruginosa*	**62.6%**	6.4%	26.7%	0
(607/1783, 34%)	(380/607)	(39/607)	(162/607)	

Definitions: CAZ, ceftazidime; C-T, ceftolozane/tazobactam; MEM, meropenem; NS, non-susceptible; TZP, piperacillin/tazobactam; S, susceptible.

TZP NS *P. aeruginosa* C-T versus MEM, *P < *0.0001; C-T versus CAZ, *P < *0.0001.

MEM NS *P. aeruginosa* C-T versus TZP, *P < *0.0001; C-T versus CAZ, *P < *0.0001.

CAZ NS *P. aeruginosa* C-T versus TZP, *P < *0.0001; C-T versus MEM, *P < *0.0001.

a The highest percentage susceptibility for a given row is indicated in bold.

**Figure 3.**
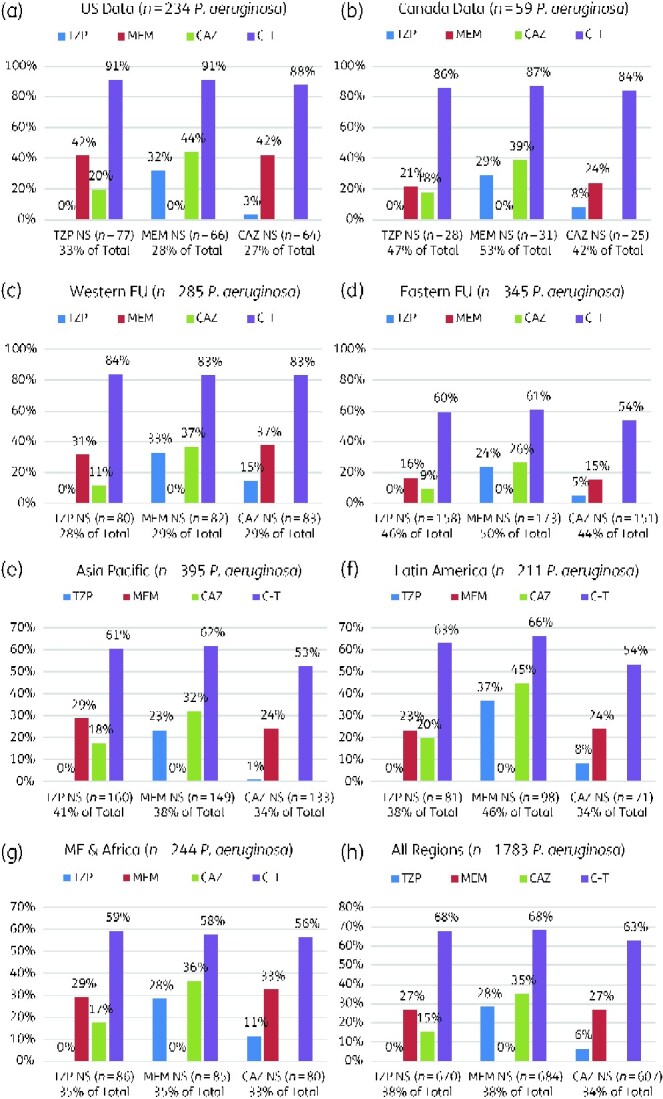
Susceptibility of *P. aeruginosa* agents when non-susceptible (NS) to first-line β-lactams, stratified by geographical region (2018 SMART Surveillance, ICU lower respiratory infections). CAZ, ceftazidime; C-T, ceftolozane/tazobactam; EU, Europe; ME, Middle East; MEM, meropenem; NS, non-susceptible; TZP, piperacillin/tazobactam.

Within the *P. aeruginosa* population, 38% displayed phenotypic non-susceptibility to piperacillin/tazobactam and meropenem, and 34% of the *P. aeruginosa* displayed non-susceptibility to ceftazidime (Figure 3 and Figure S1, available as Supplementary data at *JAC-AMR* Online). Rates of resistance to these first-line anti-pseudomonal β-lactam antibiotics varied considerably across the globe (Figure 3 and Figure S1). However, regardless of overall rates of piperacillin/tazobactam, ceftazidime or meropenem resistance, rates of co-resistance between piperacillin/tazobactam, ceftazidime and meropenem were consistent across all regions. Hence, scaling up to a carbapenem in a patient with poor response to first-line therapy may carry minimal additional benefit in terms of susceptibility. Once a Gram-negative pathogen was resistant to at least one β-lactam antibiotic, only ceftolozane/tazobactam provided additional significant activity (Figure 3 and Figure S1).

## Discussion

In critically ill patients with suspected pneumonia, prior to knowledge of organism identification and susceptibility information, physicians often prescribe empirical antibiotics based on data provided by their local antibiogram. These antibiograms traditionally display pathogen-specific antimicrobial susceptibilities to individual agents from different antibiotic classes. Although common, this method is not informative to differentiate the ability of various available empirical treatments to simultaneously cover the majority of the most likely infecting pathogens (i.e. collective susceptibility). In this study, using an international collection of isolates from all over the world, we identified that close to 80% of Gram-negative bacterial pathogens isolated from lower respiratory tract specimens from ICU patients consist of *P. aeruginosa* and Enterobacterales. Among these, ceftolozane/tazobactam provided the highest rate of susceptibility at 84%, followed by meropenem at 81%. Ceftazidime and piperacillin/tazobactam were lower at 68% and 73%, respectively. While all of these agents had minimal activity against *A. baumannii* (10%–12%), ceftolozane/tazobactam still provided the highest rate of collective susceptibility for *P. aeruginosa* plus Enterobacterales plus *A. baumannii,* which collectively comprised 92% of the Gram-negative pathogens isolated*.* While clinicians need to consider their local epidemiology when making empirical antibiotic prescribing decisions, these data would suggest the empirical use of piperacillin/tazobactam and ceftazidime warrants serious re-evaluation in treatment guidelines, given that close to one-third of patients receiving these drugs empirically would receive inappropriate therapy if *P. aeruginosa* or Enterobacterales are isolated. These international data are quite similar to a US evaluation by Sutherland & Nicolau^4^ from a 2013–14 collection of Gram-negative nosocomial respiratory isolates. Many studies have consistently shown that receipt of initially inappropriate antibiotic therapy has serious negative consequences on mortality and length of stay.^5–7^

Additional insights offered by these data include the fact that among *P. aeruginosa*, the most common Gram-negative pathogen of the cohort, resistance rates to commonly prescribed Gram-negative antibiotics for nosocomial pneumonia (meropenem, ceftazidime, and piperacillin/tazobactam) were quite consistent, and susceptibility was consistently poor, in the 62%–66% range. In contrast, ceftolozane/tazobactam susceptibility was much higher, at 87%. While rates of resistance among *P. aeruginosa* varied across geographic regions, the rate of co-resistance among older antibacterial agents was consistent. This is very important clinically because there is a perceived ‘hierarchy’ in anticipated susceptibility among *P. aeruginosa*, with increasing confidence as one moves from piperacillin/tazobactam, ceftazidime (or cefepime), meropenem, and then the newer Gram-negative antibiotics (e.g. ceftolozane/tazobactam). This cohort of international isolates suggest that this perception may be incorrect, as little additional coverage is actually gained across the older antibacterial agents and that meropenem adds little additional susceptibility. Indeed, among *P. aeruginosa* that is resistant to piperacillin/tazobactam or ceftazidime, barely one-quarter (27%) are susceptible to meropenem. Our co-resistance results appear to agree with a recently published US data for a non-urine *P. aeruginosa* collection of isolates in which co-resistance to piperacillin/tazobactam, ceftazidime and meropenem was common.^8^ Goodlet *et al*.^8^ found that among *P. aeruginosa* non-susceptible to piperacillin/tazobactam, only 45% were susceptible to meropenem; whereas ceftolozane/tazobactam susceptibility was 90% in this setting.^8^ It is not uncommon for prescribers to start therapy with piperacillin/tazobactam, ceftazidime, or cefepime and, while awaiting final susceptibility results from the laboratory in the face of a clinically deteriorating patient, ‘escalate’ therapy to meropenem or another carbapenem. Our data (Table 2) are a striking reminder that such a practice is not much of an escalation, adds little confidence to a potentially failing initial regimen, and may lead to further delays in the initiation of more-appropriate therapy. While escalation to ceftolozane/tazobactam does not ensure 100% chance of effective coverage (and thus is not a ‘perfect’ choice for *P. aeruginosa* resistant to generics) it offers a much more reliable approach.

This study has some important limitations. The data assembled and analysed were purely microbiological, with no accompanying clinical assessments or evaluations (i.e. this evaluation of collective probability of covering the most likely pathogens is only one dimension influencing antimicrobial selection). Individual patient antibiotic exposure is another very important factor not considered in this study. Therefore, the clinical implications of the differences in antimicrobial susceptibility are purely hypothetical and warrant direct clinical study for more definitive evidence as to their relevance. Nevertheless, some hints as to the discriminatory relevance between ceftolozane/tazobactam and meropenem may be extrapolated from the recently published ASPECT-NP trial.^9^ That study enrolled ventilated patients with nosocomial pneumonia, either acquired while chronically ventilated (ventilator-associated pneumonia, VAP) or who developed hospital-acquired pneumonia (HAP) severe enough to require subsequent ventilation (ventilated HAP). Among ventilated HAP patients, 28 day all-cause mortality was 24% for ceftolozane/tazobactam and 37% for meropenem (difference 13%; 95% CI, 0.2%–24.8%).^9^ In the same study, in patients who had received unsuccessful antibacterial therapy for the study episode of nosocomial pneumonia prior to enrolment, all-cause mortality at day 28 was 22.6% for ceftolozane/tazobactam versus 45% for meropenem (difference 22%; 95% CI, 3.1%–40.1%).^9^ This difference needs to be validated in a more focused clinical study but is a reminder that there is room to improve upon the treatment of patients with pneumonia in the ICU. Additional limitations include the fact that these data may not reflect those of specific hospitals and cannot substitute for a local hospital antibiogram.

In summary, this study offers the possibility that local antibiograms may consider modifying their presentation of antimicrobial susceptibility from a pathogen-specific to a site-of-infection-specific format to increase their utility in empirical settings. The latter would incorporate a collective assessment of antimicrobial susceptibility superimposed against the most common pathogens for that site of infection. Using this approach, we determined that ceftolozane/tazobactam and meropenem stood apart from other generics in providing adequate collective coverage for *P. aeruginosa* and Enterobacterales. Furthermore, these data showed that meropenem is not a ‘step up’ antibiotic in the case of failing other generic antimicrobials in patients with pneumonia in the ICU where *P. aeruginosa* is a most likely pathogen. Newer Gram-negative antibacterial agents may need to step up into a role earlier in the treatment course of these patients in order to maximize their chances of a successful clinical outcome.

## Funding

This work was supported by Merck Sharp & Dohme Corp., a subsidiary of Merck & Co., Inc., Kenilworth, NJ, USA.

## Transparency declarations

P.A.M., M.G., I.A., D.L, B.A., C.A.D., W-T.C., J.P, B.P. and M.M. are Merck Sharp & Dohme Corp., a subsidiary of Merck & Co., Inc., Kenilworth, NJ, USA employees and shareholders in Merck & Co., Inc., Kenilworth, NJ, USA. M.H. works for International Health Management Associates, Inc. (IHMA), which receives funding from Merck & Co., Inc. for the SMART surveillance program.

## Supplementary data

Figure S1 is available as Supplementary data at *JAC-AMR* Online.

## Supplementary Material

dlaa129_Supplementary_Data
