# Supplementary material for: Collective assessment of antimicrobial susceptibility among the most common Gram-negative respiratory pathogens driving therapy in the ICU
Source: JAC Antimicrob Resist. 2021 Feb 19;3(1):dlaa129. doi: 10.1093/jacamr/dlaa129 (PMC8209971; doi:10.1093/jacamr/dlaa129)
Supplement: dlaa129_Supplementary_Data [file dlaa129_supplementary_data.docx]

**Supplementary data**

**Figure S1.** Percent Susceptibility of *P. aeruginosa* Agents when Non-Susceptible (NS) to First-Line β-lactams, by Country (2018 SMART Surveillance, ICU Lower Respiratory Infections), for selected countries with at least 30 *P. aeruginosa* isolates from ICU LRI patients. CAZ, ceftazidime; C-T, ceftolozane/tazobactam; ICU, intensive care unit; LRI, lower respiratory infections; MEM, meropenem; NS, non-susceptible; TZP, piperacillin/tazobactam. (Figure continues on next page)

Eastern Europe

Western Europe

**Piperacillin/Tazobactam NS *P. aeruginosa;* Meropenem NS *P. aeruginosa;* Ceftazidime NS *P. aeruginosa***

| **Country** (Total *P. aeruginosa* from ICU RTI patients) | **NS Phenotype** (% of Total) | **C-T** % Susceptibility | **TZP** % Susceptibility | **MEM** % Susceptibility | **CAZ** % Susceptibility |
| --- | --- | --- | --- | --- | --- |
| United States (n=234) | 33%  28%  27% | 90.9  90.9  87.5 | 0  31.8  3.1 | 41.6  0  42.2 | 19.5  43.9  0 |
| Canada (n=59) | 47%  53%  42% | 85.7  87.1  84.0 | 0  29.0  8.0 | 21.4  0  24.0 | 17.9  38.7  0 |
| France (n=75) | 20%  19%  19% | 80.0  78.6  78.6 | 0  28.6  7.1 | 33.3  0  42.9 | 13.3  42.9  0 |
| Germany (n=64) | 21%  33%  23% | 92.9  95.2  93.3 | 0  47.6  6.7 | 21.4  0  26.7 | 0  47.6  0 |
| Italy  (n=52) | 44%  37%  40% | 78.3  73.7  76.2 | 0  10.5  4.8 | 30.4  0  33.3 | 13.0  26.3  0 |
| Spain  (n=35) | 29%  29%  26% | 90.0  80.0  77.8 | 0  40.0  22.2 | 40.0  0  33.3 | 30.0  40.0  0 |
| Croatia (n=47) | 55%  51%  51% | 92.3  91.7  91.7 | 0  16.7  0 | 23.1  0  25.0 | 7.7  25.0  0 |
| Hungary (n=54) | 33%  59%  24% | 100  100  100 | 0  53.1  0 | 16.7  0  15.4 | 27.8  65.6  0 |
| Lithuania  (n=33) | 39%  33%  33% | 84.6  81.8  81.8 | 0  27.3  0 | 38.5  0  27.3 | 15.4  27.3  0 |
| Russia (n=33) | 70%  79%  70% | 13.0  19.2  8.7 | 0  15.4  4.3 | 4.3  0  4.3 | 4.3  15.4  0 |
| Serbia  (n=37) | 86%  89%  89% | 21.9  21.2  21.2 | 0  3.0  3.0 | 0  0  0 | 0  0  0 |
| Turkey  (n=49) | 39%  41%  35% | 94.7  95.0  94.1 | 0  30.0  5.9 | 26.3  0  29.4 | 15.8  40.0  0 |
| Australia (n=33) | 21%  18%  18% | 85.7  83.3  83.3 | 0  33.3  0 | 42.9  0  50.0 | 14.3  50.0  0 |
| China (n=101) | 46%  48%  43% | 58.7  64.6  55.8 | 0  29.2  2.3 | 26.1  0  20.9 | 8.7  29.2  0 |
| Malaysia (n=32) | 22%  13%  19% | 57.1  75.0  50.0 | 0  75.0  0 | 71.4  0  66.7 | 14.3  50.0  0 |
| Taiwan  (n=81) | 38%  28%  23% | 93.5  95.7  89.5 | 0  30.4  0 | 48.4  0  52.6 | 38.7  60.9  0 |
| Thailand (n=38) | 47%  42%  45% | 22.2  25.0  17.6 | 0  0  0 | 11.1  0  5.9 | 5.6  0  0 |
| Argentina (n=51) | 43%  41%  37% | 81.8  81.0  78.9 | 0  19.0  5.3 | 22.7  0  26.3 | 18.2  33.3  0 |
| Brazil (n=42) | 31%  48%  19% | 84.6  90.0  75.0 | 0  50.0  0 | 23.1  0  25.0 | 38.5  70.0  0 |
| Mexico (n=39) | 26%  38%  15% | 90.0  93.3  83.3 | 0  66.7  16.7 | 50.0  0  66.7 | 50.0  86.7  0 |
| Israel (n=51) | 29%  20%  31% | 86.7  80.0  87.5 | 0  40.0  31.3 | 60.0  0  68.8 | 26.7  50.0  0 |
| Tunisia (n=52) | 27%  42%  25% | 42.9  63.6  46.2 | 0  50.0  15.4 | 21.4  0  23.1 | 21  54.6  0 |
| **All Countries (n=1783)** | 38%  38%  34% | 67.8  68.1  62.6 | 0  28.2  6.4 | 27.7  0  26.7 | 15.2  34.9  0 |

Middle East and Africa

Latin America

Asia Pacific
